# Distraction osteogenesis in dog with a tooth-borne device: Histological and histomorphometric analysis

**DOI:** 10.4317/jced.56491

**Published:** 2020-01-01

**Authors:** Francisco Vale, Inês Francisco, João Cavaleiro, Francisco Caramelo, Adriana Guimarães, João Brochado

**Affiliations:** 1 DDS, MSc. PhD. Program Director and Head of Department, Institute of Orthodontics, Faculty of Medicine, University of Coimbra, Portugal; 2 DDS, MSc. Assistant Professor, Institute of Orthodontics, Faculty of Medicine, University of Coimbra, Portugal; 3 DDS, MSc. Postgraduate in Orthodontics, Institute of Orthodontics, Faculty of Medicine, University of Coimbra, Portugal; 4 PhD. Professor, Institute of Clinical and Biomedical Research of Coimbra (iCBR), Faculty of Medicine of the University of Coimbra, Portugal; 5 DDS, MSc. Assistant Lecturer, Histology and Embryology Institute, Faculty of Medicine, University of Coimbra, Portugal

## Abstract

**Background:**

The distraction osteogenesis (DO) is the biological process of new bone formation between the surfaces of bone segments gradually separated by incremental traction. However, the lack of solid experimental studies using the tooth-borne distractor does not allow comparing this technique with the classical procedures. This study aimed to establish the effect of two different activation protocols in new bone formation, with a new intraoral tooth-borne device for dog mandibular distraction osteogenesis.

**Material and Methods:**

Nine beagle dogs were split into 3 similar groups, Group A the control, Group B subjected to two daily activations of 0.5 mm and Group C subjected to a single daily activation of 1 mm. The distraction period was 10 days followed by a 12 weeks consolidation period. Samples where then processed and embedded in methylmethacrylate and ground to a thickness of 20µm. Toluidine blue stains were done on all specimens and histological and histomorphometric evaluation of bone tissue formed within distraction gap was performed. The statistical analysis in this manuscript was performed with IBM®-SPSS® v.20 statistics software and R software version 3.1.0. The level of significance adopted was 5 % (α=0.05).

**Results:**

No statistically significant difference was detected by histomorphometric evaluation between the two experimental groups in what concerns the bone volume. However, significant differences were found in the coefficients of variation between the medial and buccal areas, and the buccal and lingual areas.

**Conclusions:**

This study shows that the mandible can be lengthened successfully using a tooth-borne distractor. Moreover, it suggested that a decrease from once to twice-daily activations might negatively change the quality and structure of newly formed bone and prompt it to instability.

** Key words:**Retrognathia, bone regeneration, osteogenesis, distraction.

## Introduction

The distraction osteogenesis (DO) is the biological process of new bone formation between the surfaces of bone segments gradually separated by incremental traction (1,2). Traction generates tension within the callus and stimulates new bone formation parallel to the vector of distraction. In fact, DO is based upon the “tension-stress principle” that uses the physiologic mechanisms of the human body to heal and reconstruct in a true tissue engineering manner (1,3).

Mandibular DO results from a gradual mechanical traction that is applied at the osteotomy site created in the jaw. This controlled mechanical stress promotes and maintains angiogenesis and osteogenesis as well as the growth of surrounding soft tissues between the two osteotomy edges (4,5). Although the rate of DO can influence the overall process, few experimental studies assessed the effect of the activation protocols on the quality and quantity of newly formed bone tissue in the manipulated mandible. More recently, new and more conservative approaches are being designed looking mainly to increase patients compliance and comfort without losing the effectiveness of the procedure. In this regard, the development of intra-oral and tooth-borne distractors emerged, thus allowing mandibular DO without surgical interventions, favorable orientation of the distraction force vector and increased treatment predictability. However, the lack of solid experimental studies using the tooth-borne distractor does not allow the formulation of any definitive conclusion regarding its preferential use when compared to the classical procedures.

## Material and Methods

This study aimed to assess the efficacy of the tooth-borne distraction appliance through the evaluation of the quantity and quality of the newly formed bone after the consolidation period. To this end, two different distraction activation protocols were used and the results were evaluated using histological and histomorphometric techniques.

**Figure 1:**
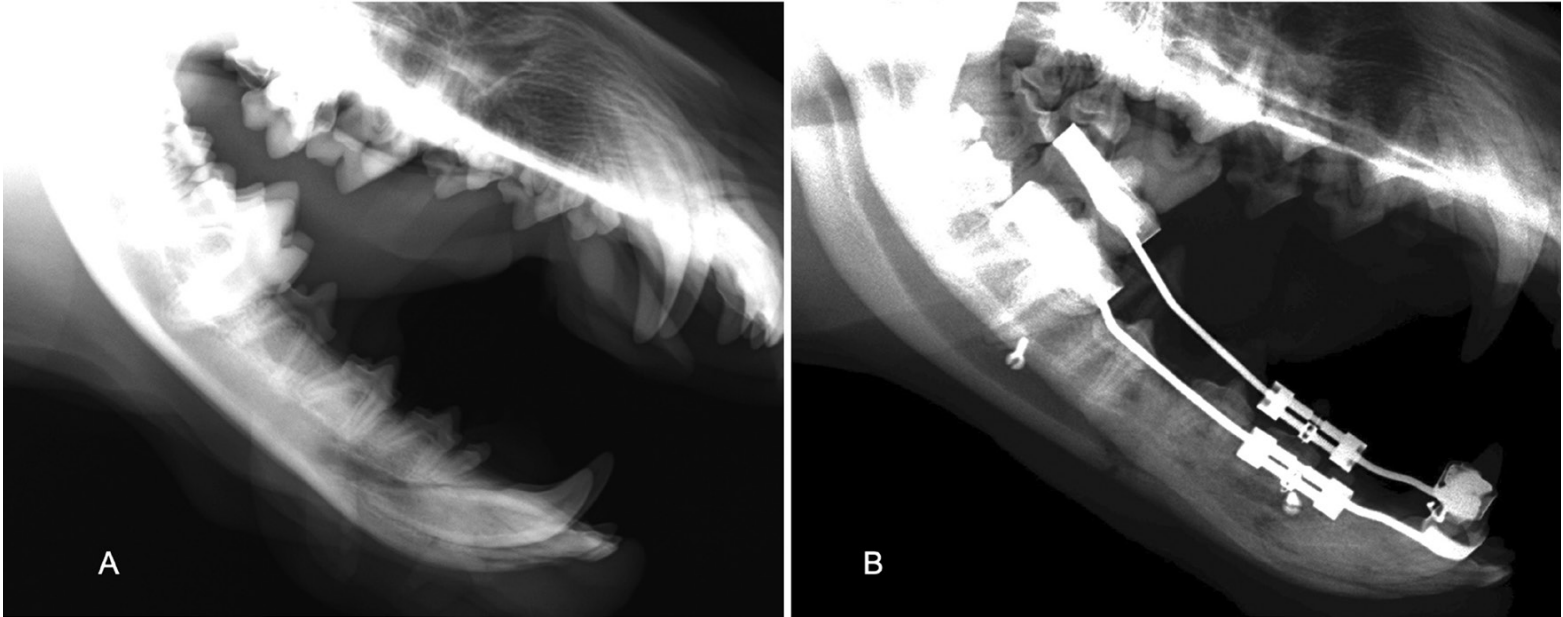
Lateral Cephalogram before surgery (A) and after consolidation period (B).

Nine skeletally mature conditioned male Beagle dogs, approximately 1 year old and weighing 15 to 18 kg, were used in this study. The laboratory-manufactured mandibular distractor (Fig. 1) employed was uniplanar and unilateral and consisted of a stainless-steel disjunction screw (Variety SP® DENTAURUM GmbH & Co., Ispringen, Germany) adapted and welded to orthodontic bands through two 1.2 mm diameter connector bars, with universal silver-based and cadmium-free soldering of 0.1 mm in diameter (Produites Dentaires SA, Vevey, Switzerland). The working mechanism of the screw is a rotation-pressure type, allowing the demultiplication of the effort for the elongation, with a maximum expansion of 12 mm. The rotational movement of 3600 around its axis produces a translational movement (sagittal elongation) of 1.0 mm. A single activation allows a rotation of 900 (1/4 of a turn), producing a 0.25 mm elongation. The device also has a brake system that prevents the accidental return of the activation.

Dogs were split into 3 experimental groups, each containing 3 animals. Group A constituted the control group that experienced no surgical procedures. The remaining six animals underwent a bilateral midbody osteotomy performed between the third and fourth premolar, preserving the mandibular nerve and the integrity of the lingual periosteum, followed by the insertion of a tooth-borne distractor in each hemimandible after confirming bone mobility. Following a latency period of 7 days recommended by McCarthy and collaborators6, these six animals were divided into two experimental groups: Group B, in which mandibles were subjected to two daily activations of 0.5 mm, with an interval of 12 hours, for 10 days; and Group C that were subjected to a single daily distraction of 1 mm, also for 10 days. Following a total length of distraction of 10 mm in both experimental groups, all devices were properly blocked and a consolidation period of 12 weeks took place. Subsequently, the animals were sacrificed using intravenous sodium pentobarbital (100mg/kg), and the hemimandibles were trimmed and fixed in 70% ethanol for 1 week. The samples where processed and embedded in methylmethacrylate, sectioned and ground to a thickness of 20 µm on an Exakt Cutting-Grinding System (Exakt, Hamburg, Germany). Toluidine blue stains were performed in all specimens and histological evaluation of bone tissue formed within distraction gap was then executed.

Each sample was analyzed using a light microscope (Nikon® SMZ 1500, Tokyo, Japan) for histomorphometric evaluation of newly formed bone using the Bioquant Osteo® 2012 software (Bioquant® - Image Analysis Corporation, Nashville, EUA). On each image, a grid containing three horizontal rows (coronal, central, apical) of 3 regions of interest (ROI) each was placed over the buccal, middle and lingual planes of the regenerate area, for a total of 9 ROIs on each hemimandible section.

The variables were described resorting to central tendency and dispersion measures suited to the type of variable. Differences between independent groups were assessed with the Mann-Whitney test, after normal distribution confirmation with the Shapiro-Wilk test. In order to evaluate the dispersion of values between groups, Levene test was applied to the variation of coefficient of the different groups. The statistical analysis in this manuscript was performed with IBM®-SPSS® v.20 statistics software and R software version 3.1.0. The level of significance adopted was 5 % (α=0.05)

This animal model study was performed according to the rules and regulations of the Portuguese National Authority for Animal Health, with approval protocol number 04200000002012.

## Results

The overall results of histomorphometric analysis are illustrated in  Table 1.  Table 2 depicts new bone formation for each evaluated ROI.

**Table 1:**
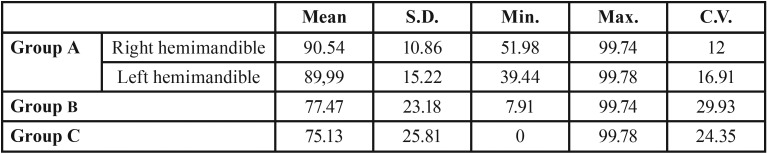
Results for histomorphometric bone quantification. The arithmetic mean (Mean), standard deviation (SD), method error (ME), minimum (Min), maximum (Max), and coefficient of variation (CV) are given.

**Table 2:**
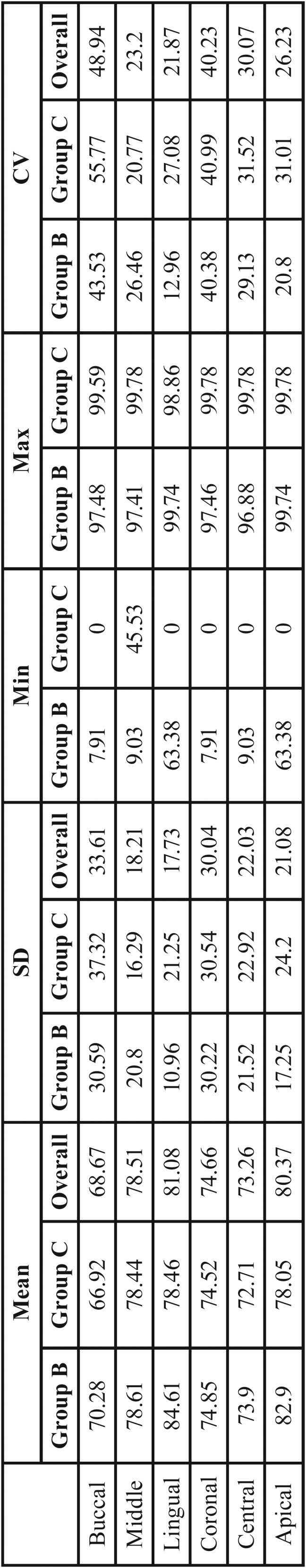
Results for histomorphometric evaluation of new bone formation in each region of interest (ROI). The arithmetic mean (Mean), standard deviation (SD), method error (ME), minimum (Min), maximum (Max), and coefficient of variation (CV) are given.

Group A

**Figure 2:**
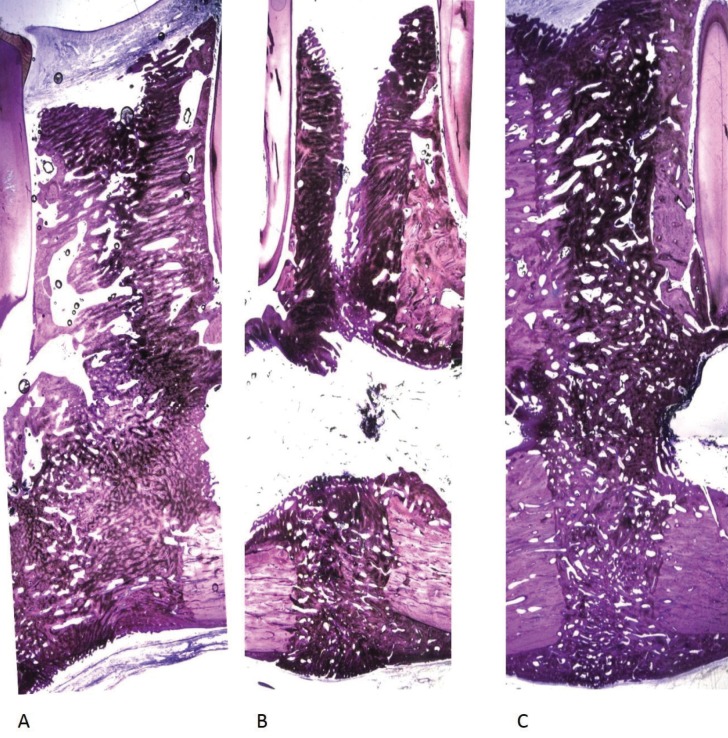
8 week consolidation regenerate (7,5x). Histologic section of regenerate (distraction gap) with (A) buccal aspect of bony trabeculae extending up to half of the total width of distraction gap; (B) new bone formation located at the host bone margins with fibrovascular tissue from the center of the gap up to alveolarcanal; (C) lingual/cortical areas completely obliterated by bony trabeculae originating from the native bone.

As expected, only bone tissue was observed in the untreated control group (Fig. 2). Bony trabeculae occupied 90.25 ± 13.18 % of the space.

Group B

After the consolidation period, distraction gap (DG) of samples from group B were consistently bridged by a remarkable amount of new bone (77.47±23.18%). The production of this newly formed woven bone started from the local host surface inwards to the distraction gap, showing a higher degree of maturation and organization in more peripheral areas. Moreover, a tendency to be aligned in the direction of the distraction forces was also observed.

Moving towards the central area of distraction, least differentiated trabecular bone with significant remodeling signs was also observed, along with some areas of immature bone tissue covered with recently synthesized lamellar bone. However, areas of new bone formation were also found adjacent to the inferior cortex, filling that big space between the outer surface of the cortical bone and the periosteum - bone callus. This aspect was mostly evident in the basilar edge of the mandible, particularly in the buccal samples.

**Figure 3:**
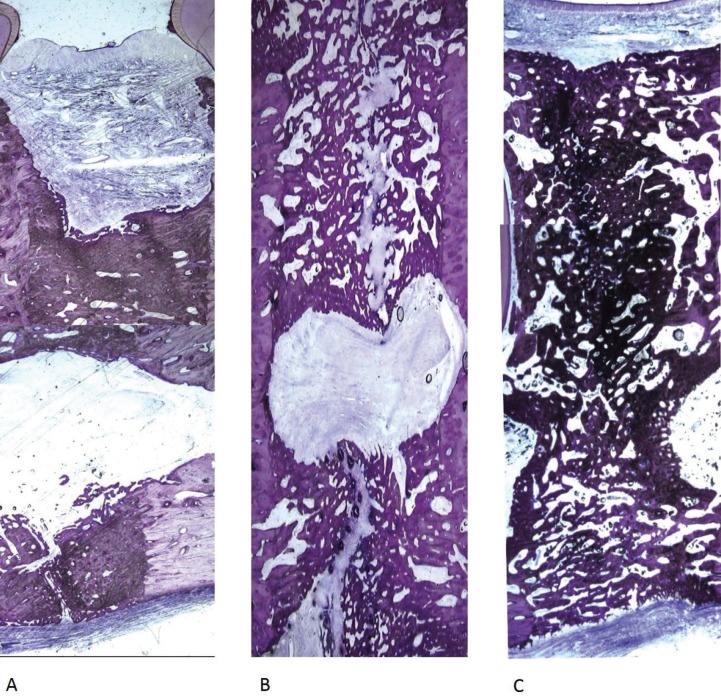
8 week consolidation regenerate (7,5x). Histologic aspect of the distraction gap showing discrepancies in regenerate bone consolidation along the buccal-lingual axis. The fibrous interzone was usually wider and bigger at the buccal (A) than at the central (B) or lingual (C) extents of the regenerate. At the central aspect can be noted the presence of newly formed woven bone and some areas of cartilage/fibrocartilage tissue.

Assumed the orientation of the histological sections performed for each hemimandible, the presence of cartilage matrix was commonly observed in the center of the distraction gap, particularly in the buccal samples (Fig. 3). Evaluation of the individual ROIs of buccal areas demonstrated that bony trabeculae occupied 70.28 ± 30.59 % of the space, while samples from middle and lingual surfaces were characterized by a great density of newly formed bone, with a complete obliteration of the DG in almost all sections. The average trabecular bone area of the middle and lingual area was 78.61 ± 20.8 % and 84.61 ± 10.96 %, respectively.

In a vertical perspective (apical to coronal), heterogeneity was also found in the distribution of newly formed bone tissue. Indeed, a higher density of trabecular bone was present in the apical portion (82.90 ± 17.25 %) and remarkably in the central zone (73.90 ± 21.52 %), as opposed to what was observed in the coronal region (74.85 ± 30.22 %).

Although a solid and uninterrupted structure of bone completely filled the hemimandibles’ gap, one hemimandible showed an incomplete union and the empty space was filled with a thin layer of fibrous or cartilaginous tissue (Fig. 3).

Group C

Histomorphometric analysis of samples from group C revealed 75.13 ± 25.81 % of new bone regenerate in the DG. However, the central zone of distraction gap depicted correspondingly less mature repair tissue ranging from hyaline cartilage and/or fibrocartilage to fibrous tissue. In addition, the occurrence of endochondral bone formation in the fibrocartilage islands was frequently observed, as were focal regions of bone-surrounded chondrocytes, suggesting transchondroid bone formation.

An axis of cartilage matrix was also commonly observed in the central region of many bone trabeculae, and histological sections additionally revealed some degree of heterogeneity in the distribution of newly formed bone, both in apical-coronal direction and in buccal-lingual. Differences were also detected in the microscopic organization of bone tissue, particularly in the vicinity of the mandibular canal wall.

In the buccal area the average of newly formed bone was only 66.92 ± 37.32 %, in contrast to middle and lingual areas in which trabecular bone occupied 78.44 ± 16.29 % and 78.46 ± 21.25 %, respectively. Moreover, histomorphometric analysis also revealed heterogeneity in the distribution of the newly formed bone in apical-coronal direction with 74.52 ± 30.54 % bone formation in the coronal section, 72.71 ± 22.92 % in the central area and 78.05 ± 24.20 % in the apical portion of the distraction gap.

Areas of cartilage matrix were more often observed in the central space of the buccal surface when compared to the medial or lingual regions.

Histomorphometric comparison of the total newly formed bone between groups B and C 

Comparison of the amount of newly bone formed tissue in the DG between the experimental groups B and C revealed no statistically significant differences (U=2233.5; Z=-0.288; *p*=0.773). Also, no statistically significant differences were found in the coefficient of variation between the groups B and C [F(1.134)=1.234; *p*=0.269] . However, although not statically significant, qualitative analysis of the DG have shown a greater number of non-union situations in sections from group C, in addition to the presence of a higher number of areas filled by cartilaginous tissue.

Histomorphometric comparison of the total newly formed bone between buccal, medial and lingual regions

Evaluation of individual ROIs revealed that bony regenerate occupied up to 68.67 ± 33.61 % in the buccal, 78.51 ± 21.00 % in the medial and 81.08 ± 17.73 % in the lingual ROIs. However, a statistically significant difference was found in the coefficients of variation between the medial (23.20%) and buccal (48.94%) areas ((F(1,87)=28.222; *p*<0.001), and between the buccal (48.94%) and lingual (21.87%) areas ((F(1,89)=35.702; *p*<0.001)).

Histomorphometric comparison of the total newly formed bone between coronal, central and apical regions

Bony regenerate occupied up to 74.66 ± 30.04 % of the coronal region, 73.26 ± 22.03 % of the central and 80.37 ± 21.08 % of the apical region ROIs. No statistically significant differences were found between the ROIs (F(2,133)=1.099; *p*=0.336) and no differences were found between the coefficients of variation of coronal and central zones (F(1,86)=0.928; *p*=0.338), apical and central zones (F(1.91) = 1.023; *p*=0.314) and coronal and apical zones (F(1,89)= 1.194; *p*=0.077).

## Discussion

Bone regeneration during DO is a unique and powerful form of endogenous bone tissue engineering. In fact, the mechanical environment created by the gradual distraction strongly activates bone tissue formation mainly by intramembranous ossification. However, similarly to fracture healing, DO is highly susceptible to the mechanical factors associated with the length of interfragmentary movement, particularly the distraction rate and frequency during DO (7), which is itself believed to govern the type of tissue and phenotypic differentiation of the cells within the DG (7-9).

There is currently great consensus establishing the optimal rate of distraction in the 1 mm/day (3-14). However, as to the frequency of activation, opinions are more divergent and clinicians tend to follow Ilizarov rationale, according to which a greater frequency provided a better outcome, for the same rate of distraction (2). This way, aiming a 1mm/day rate, twice-daily 0,5 mm activations are more commonly used than a single 1 mm daily activation (10-14).

Increased frequencies of activation are strongly correlated with the acceleration of bone regeneration (8-14) and a shorter consolidation time.8 In agreement, the present results indicated that a better bone consolidation and higher amounts of lamellar bone tissue were attained using a twice-daily activations protocol (group B). Moreover, cases of non-union were more often observed on group C where a daily activation protocol was implemented. This may have been associated with a higher degree of mechanical instability allowed by this protocol, which promoted excessive bone movement and more microvascular disruption, thus compromising osteogenesis. In support of these hypotheses, Marsell and colleagues observed that indirect bone healing, which permits some degree of motion between the bone fragments, stimulates intramembranous and endochondral bone formation (16). Additionally, it comprises a more controlled inflammation environment and an earlier development of a stabilizing external cartilage callus, thus permitting a quicker repair of the distraction gap. Nonetheless, the group of Evans showed that excessive motion between the bone fragments might delay the healing process and eventually result in the non-union of the bone tops (17). An inadequate consolidation period may also cause discontinuity or subsequent shrinkage of the regenerated bone (18).

The discussion on the mechanism behind ossification in DO is still ongoing (2,19-21). Komuro and cols. reported that in rabbit mandible new bone can be formed by either intramembranous or endochondral ossification, although Sawaki group argued that some fibrocartilage islands are indispensable for intramembranous ossification (22,23). Yasui and collaborators (24), on the other hand, identified a transchondroid ossification mechanism in a rat model of long bone lengthening, where following cartilage formation (possibly due to the low oxygen tension), there is a direct transformation of chondroid tissue into bone (24,25).

Due to the fact that in the present study the histological evaluation was only performed at the end of the consolidation period, the mechanism behind bone formation was impossible to access with certainty. However, remains of cartilage and chondroid tissue were found in some mandibles, indicating that at least endochondral and/or transchondroid ossification were involved.

Yasui and collaborators (24) also suggested that the cartilage formed during DO is observed at the level of the periosteum but not between the tops of the cortices within the distraction gap. Still, the results attained in the present study contradict those observations, as in both experimental groups large areas of woven bone of periosteal origin were evident in a sub-periosteal location, thus representing an unquestionable process of intramembranous bone formation.

Regardless of the regenerative mechanism involved, higher numbers of cartilage islands were found at the center of distraction gap in the mandibles that failed to regenerate, in buccal and in close proximity to the mandibular nerve (either slightly inferior or superior to the inferior alveolar canal). This may have been related to local environment, more specifically with the intraosseous location of the mandibular nerve, which allows the interaction of the DO machinery with neuro-active molecules and peptides. On the other hand, the area of cartilage formation laid within the neutral axis of the regenerate where tensile forces are minimal, which may also have disfavored regeneration (5,25-27). It may have also happened that the expansion capacity of the stromal tissue, particularly that of the associated capillaries, failed to meet the demands of a twice-daily activation protocol, thus creating a hypoxic environment that rather promoted the formation of cartilaginous tissue (28-30). It is important to point out that these three mechanisms rather than alternative may have been concurrent to the same endpoint.

Whenever formed, the new bone varied in terms of amount and location in all 3 dimensions. The histological findings showed more evident variances along the vertical height of the regenerate, with fibrous tissue tendentially being retained in the crestal regions. This was most probably due to an inadequate amount of interradicular host bone in the crestal region on either side of the osteotomy line. In agreement, osseous defects were rarely observed in the cortical regions where the host bone was relatively thick and no tooth roots were present.

Finally, discrepancies in regenerate bone consolidation were observed in the buccal-lingual axis. The fibrous interzone was usually wider and bigger at the buccal region compared to either the center or the lingual extents of the regenerate. Lingual regions never had an osseous defect and were always the first place where the interzone was obliterated. In fact, this cortical region depicted the higher rate of bone formation. However, it is essential to consider that since there was no periosteum injury at this level, the surgical trauma was much smaller as opposed to what happened in both the buccal and cortical surfaces.

Altogether, the attained results allow us to conclude that the tooth-borne distractor permitted a correct lengthening direction and the creation of the desirable stress force necessary to the osteogenic process. In fact, its use showed several benefits, e.g. the absence of surgical interventions for the placement or the removal of the device (being the only osteotomy surgery); favorable orientation of the distraction force vector, increased patient comfort and compliance, increased treatment predictability and decreased morbidity. Furthermore, this study provided interesting preliminary data supporting the further investigation of this method as more definitive conclusions about its use are needed to help adapting and optimizing DO treatment protocols.

## Conclusions

This study shows that the mandible can be lengthened successfully using a tooth-borne distractor. Moreover, it suggested that an increase from once to twice-daily activations improve the quality and structure of newly formed bone and prompt it to better stability.
